# Continued cognitive-behavior therapy versus sertraline for children and adolescents with obsessive–compulsive disorder that were non-responders to cognitive-behavior therapy: a randomized controlled trial

**DOI:** 10.1007/s00787-014-0613-0

**Published:** 2014-09-20

**Authors:** Gudmundur Skarphedinsson, Bernhard Weidle, Per Hove Thomsen, Kitty Dahl, Nor Christian Torp, Judith B. Nissen, Karin Holmgren Melin, Katja Hybel, Robert Valderhaug, Tore Wentzel-Larsen, Scott N. Compton, Tord Ivarsson

**Affiliations:** 1 Center for Child and Adolescent Mental Health, Eastern and Southern Norway, Gullhaugveien 1-3, 0484 Oslo, Norway; 2 Regional Center for Child and Youth Mental Health and Child Welfare, Norwegian University of Science and Technology, Klostergate 46, 7491 Trondheim, Norway; 3 Research Department, Center for Child and Adolescent psychiatry, Aarhus University Hospital, Skovagervej 2, Indgang 81, 0824 Risskov, Denmark; 4 Department of Child and Adolescent Psychiatry, BUP Specialmottagning, Queen Silvia’s Children’s Hospital, Sahlgrenska University Hospital, 41685 Gothenburg, Sweden; 5 Duke University Medical Center, DUMC 3527, Durham, NC 27710 USA

**Keywords:** Cognitive-behavior therapy, Selective serotonin reuptake inhibitors, Sertraline, Obsessive–compulsive disorder, Children and adolescents, Treatment outcome

## Abstract

Expert guidelines recommend cognitive-behavior therapy (CBT) as a first-line treatment in pediatric obsessive–compulsive disorder (OCD) and the addition of selective serotonin reuptake inhibitors when CBT is not effective. However, the recommendations for CBT non-responders are not supported by empirical data. Our objective was to investigate the effectiveness of sertraline (SRT) versus continued CBT in children and adolescents that did not respond to an initial course of CBT. Randomized controlled trial conducted in five sites in Denmark, Sweden and Norway, 54 children and adolescents, age 7–17 years, with DSM-IV primary OCD were randomized to SRT or continued CBT for 16 weeks. These participants had been classified as non-responders to CBT following 14 weekly sessions. Primary outcomes were the CY-BOCS total score and clinical response (CY-BOCS <16). The study was a part of the Nordic Long-Term OCD Treatment Study (NordLOTS). Intent-to-treat sample included 50 participants, mean age 14.0 (SD = 2.7) and 48 % (*n* = 24) males. Twenty-one of 28 participants (75 %) completed continued CBT and 15 of 22 participants (69.2 %) completed SRT. Planned pairwise comparison of the CY-BOCS total score did not reveal a significant difference between the treatments (*p* = .351), the response rate was 50.0 % in the CBT group and 45.4 % in the SRT group. The multivariate *χ* ^2^ test suggested that there were no statistically significant differences between groups (*p* = .727). Within-group effect sizes were large and significant across both treatments. These large within-group effect sizes suggest that continued treatment for CBT non-responders is beneficial. However, there was no significant between-group differences in SRT or continued CBT at post-treatment.

## Introduction

Obsessive–compulsive disorder (OCD) is a chronic and disabling disorder [33, 37, 41] with a population prevalence rate between 1 and 3 % [11, 32, 37, 50]. One-third to one half of adults with OCD report that the onset of their symptoms began in childhood [34]. OCD is associated with significant functional impairment [49] and ranked as the 10th leading worldwide cause of years lived with disability of all somatic or psychiatric disorders [25]. Since the 1980s, people with diagnosable OCD have been identified earlier and treatments for OCD have become increasingly effective [2, 13, 54]. These advancements will likely reduce the chronicity of OCD [23, 41, 53] and its total economic cost to individuals and society.

Although current treatments for pediatric OCD are effective, not all patients experience sufficient benefit [2, 54]. In one of the largest randomized controlled trial (RCT) in pediatric OCD, the POTS Study [30], children and adolescents were randomized to four treatment conditions: cognitive behavioral therapy (CBT), sertraline (SRT), the combination of both (COMB), and pill placebo (PBO). After 12 weeks of acute treatment, clinical remission [defined as a Children’s Yale–Brown Obsessive Compulsive Scale (CY-BOCS) of <11] was reported in 21, 39, and 54 % of participants randomized to SRT, CBT, and COMB, respectively [30]. While these results are encouraging, it also suggests that almost 50 % of children and adolescents who receive evidence-based treatments will fail to show an adequate response. Moreover, follow-up studies of participants in randomized clinical trials suggest that 40 % of children and adolescents with OCD will continue to meet diagnostic criteria for OCD 1–15 years following the end of acute treatment [23, 41] and an additional 20 % will have clinically significant residual symptoms [41]. The development of interventions for treatment-refractory OCD is therefore critically important.

Current expert guidelines recommend CBT as the first-line treatment for children and adolescents with OCD [26]. If CBT is not sufficiently effective, medication augmentation [specifically, the selective serotonin reuptake inhibitors (SSRIs)] is recommended [1, 26]. However, the sequence of treatment implicit within these guidelines (namely, start with CBT and if the patient does not respond, then augment with an SSRI) is based on expert consensus, not empirical evidence. They also lack clinically useful details such as how many sessions of CBT should be provided before it is considered inadequate.

Although expert guidelines recommend medication augmentation when CBT is not effective, continued CBT without medication augmentation may also be a viable option. In most efficacy studies, CBT has been conducted for a limited period of time. The number of weekly CBT sessions commonly ranges between 10 and 16 [54]. It is not known if CBT response rates would be higher if more sessions were provided. It seems reasonable that CBT treatment of longer duration may increase response rates, particularly in severe cases in which more time may be needed before the benefit can be seen [18]. This question, however, has not been addressed by clinical researchers.

In clinical settings, clinicians often face the decision of whether to provide additional sessions of CBT for patients who show a partial response or recommend switching to an alternative treatment. Results from a randomized controlled trial (RCT) in adults with OCD suggest that switching to fluvoxamine over continued CBT in non-responders may be more effective [51]. However, there are no similar empirical studies in pediatric populations. The question of whether medication is more effective than continued CBT in pediatric OCD remains unanswered.

This gap in the literature was one of the reasons that led to the Nordic Long-Term OCD Treatment Study (NordLOTS). NordLOTS is a multi-national, multi-step trial designed in part to evaluate the relative efficacy of continued CBT or switch to SRT among children and adolescents with OCD who were rated as non-responders to an initial course of individual CBT [18, 45]. In NordLOTS, 269 children and adolescents with OCD were offered 14 sessions of weekly individual exposure-based CBT, as first step of clinical care (Step 1). Non-responders to Step 1 were then randomized to two alternative Step 2 treatments: (1) continued CBT or (2) SRT for additional 16 weeks (Step 2). All treatments were delivered in two specialized OCD clinics and 17 community treatment centers in three countries (Denmark, Norway, and Sweden). Clinical response, assessed at the end of Step 1 treatment by independent evaluators (IEs), was defined as a CY-BOCS total score ≤15. Based on this criterion, 27.4 % (*n* = 66) of the original 269 participants in the NordLOTS study were determined to be non-responders to CBT [47] and eligible for Step 2 treatments. This article reports the results of Step 2 outcomes. Based on findings reported in [51], it was hypothesized that SRT would be superior to continued CBT in this population.

## Methods

### Study design

The rationale, design, and methods of the NordLOTS trial have been described in detail elsewhere [18, 45, 47]. A brief overview is provided here. NordLOTS included three main treatment steps. For Step 1, participants were recruited through referrals from community mental health centers, general practitioners, child mental health specialists or from parents/relatives who read information about the study trial on the internet. A total of 19 clinics in Denmark, Norway, and Sweden participated in the study within the following five main geographic areas: (1) Eastern and Southern Norway, (2) Central Norway, (3) Gothenburg, Sweden, (4) Stockholm, Sweden, and (5) Aarhus, Denmark. Participants included in Step 1 received 14 individual weekly treatment sessions of CBT. Experienced child and adolescent clinical psychologists, psychiatrists, or other specialized mental health professionals provided treatment. Therapists underwent 2 weeks of training in the NordLOTS treatment manual for pediatric OCD. To ensure understanding of the study and competent delivery of treatment, clinicians were required to treat at least two non-study patients under close supervision before being allowed to treat study participants. Clinicians received monthly group supervision within their respective sites.

Sessions were audiotaped and treatment fidelity for Step 1 was evaluated by an experienced CBT therapists involved in the project. Sessions were evaluated across three dimensions: (1) treatment adherence, (2) treatment competency, and (3) quality of the therapeutic relationship. Ninety-five percent of the audiotapes evaluated received a rating of “good” to “very good” [45]. Eleven child therapists in seven clinics provided continued CBT while eight child psychiatrists in five clinics provided the pharmacological treatment. The trial was approved by the Norwegian, Swedish and Danish Committees for Medical and Health Research Ethics and the Medical Products Agencies. The project was registered in Current Controlled Trials (www.controlled-trials.com ISRCTN66385119). The Center for Child and Adolescent Mental Health (RBUP), in Oslo, served as the coordinating center and the data management center. Informed consent was provided by parent(s) or guardian(s) and by children 11 years or older. No industry funding was provided. Funding was applied for at each national site so the total study received funding from national funders as well as some central funding. A list of all funding sources has been published elsewhere [45].

To randomize participants to Step 2 treatments, a block-wise stratified randomization procedure was applied. Gender and the presence of a tic disorder were used as stratification variables, as gender and tics have been shown to moderate treatment outcomes [10, 22]. To ensure that randomization could not be predicted in advance, the randomization procedure was centralized at the coordinating center (RBUP) in Oslo.

### Study participants

All NordLOTS Step 1 participants who were rated as CBT treatment non-responders (CY-BOCS ≥16), and who were willing to accept one of the two randomized treatments (i.e., either continued CBT or SRT), were eligible to participate in NordLOTS Step 2. The cut-off CY-BOCS score was chosen as it represents participants with moderate or severe OCD. Due to ethical issues we wished to avoid treating participants with only mild OCD with sertraline. Being above this cut-off score includes the possibility of a treatment response in terms of major symptom reduction and does not necessarily reflect that participants did not respond to treatment in Step 1. The term “non-responder” was chosen for its convenience. However, we estimated the agreement between CY-BOCS <16 and 30 % or more reduction on the CY-BOCS from baseline to week 13. The kappa showed substantial agreement (0.75) as 7.1 % (*n* = 17) had 30 % or more reduction, but still had 16 points or more on the CY-BOCS. Likewise, only 2.2 % (*n* = 6) participants had 15 or less points on the CY-BOCS without obtaining 30 % or more reduction on the CY-BOCS [47]. The inclusion and exclusion criteria of Step 1 and any additional criteria relevant to Step 2 are summarized below.

#### Inclusion and exclusion criteria

Children and adolescents, between the ages of 7 and 17 years at the beginning of Step 1, who were rated as non-responders (CY-BOCS ≥16) at the end of Step 1 treatment and accepted randomization were eligible to participate in Step 2. The diagnosis of OCD [4] and the presence of comorbid disorders was confirmed at baseline assessment. To enhance generalizability and match the sample of children commonly seen in outpatient settings, children with psychiatric co-morbidities were allowed to participate in Step 1 and Step 2 as long as the comorbid disorder did not have a higher treatment priority (e.g., psychosis and severe depression). Exclusion criteria were by design kept to a minimum. Children in Step 1 (and therefore, Step 2) were excluded if they had an IQ below 70, could not speak or understand the language in the country where the study was conducted, had a psychiatric comorbidity that made participation in NordLOTS clinically inappropriate (for example, primary anorexia nervosa; anorexia in partial remission where OCD had become the residual and primary disorder was permitted), depression with suicidality that demanded treatment, psychosis, or autism spectrum disorders. However, PDD-NOS was allowed if symptoms of OCD were more impairing. Participants receiving treatment for OCD with either CBT or medication currently or within 6 months of the start of the study were also excluded. Two additional exclusion criteria were applied for participants in Step 2: (1) post-pubertal girls who were sexually active and who did not accept or tolerate adequate contraceptive methods were excluded and (2) post-pubertal girls with a positive pregnancy test.

### Measures

The Schedule for Affective Disorders and Schizophrenia for School-Age Children Present and Lifetime Version (K-SADS-PL) [19] was used for diagnostic assessments at baseline (Step 1. The Children’s Yale-Brown Obsessive Compulsive Scale (CY-BOCS) [39] was used for assessment of scalar treatment response. The CY-BOCS is a clinician administered instrument that evaluates obsessions and compulsions separately on time consumed, distress, interference, resistance, and control. It yields separate severity scores for obsessions and compulsions (0–20) and a composite severity score (0–40). CY-BOCS assessments were completed at baseline, weeks 7 and 13 in Step 1 and weeks 22 and 30 in Step 2. The primary outcomes were the CY-BOCS total score and binary clinical response defined as CY-BOCS ≤15. A cut-off score of ≤15 has been used in previous treatment studies for identifying treatment response, including a number of pharmacological studies [14, 35, 44]. Secondary outcomes were 30 % or more reduction on the CY-BOCS from end of Step 1 treatment (week 13) to end of Step 2 treatment (week 30). Clinical remission was defined as week 30 CY-BOCS ≤10. CY-BOCS interviews were videotaped or audiotaped, and inter-rater agreement (reliability) was assessed independently from a random sample of 12.8 % of the interviews. The intra-class correlation coefficient for the CY-BOCS was 0.92 for the total score and 0.94 and 0.87 for obsessions and compulsions, respectively [47].

The Child Obsessive–Compulsive Impact Scale (COIS-R) is a 33-item self-report questionnaire designed to assess the impact of OCD symptoms on the psychosocial functioning of children and adolescent in home, social, and academic environments [29]. Each item is scored on a 4-point Likert scale (0 = not at all, 1 = just a little, 2 = pretty much, and 3 = very much). Both parent and youth versions were used. Both versions have shown moderate to high internal consistency, for children *α* = 0.78 and parents *α* = 0.92 [29].

Adverse events (AEs) were carefully assessed in participants treated with SRT. AEs were defined as any harmful and undesired change in the child’s condition since starting Step 2 treatment. Life-threatening events, hospitalization, disability or permanent damage were classified as serious adverse events (SAEs), in accordance with US Food and Drug Administration guidelines [48]. At Step 2 baseline and every visit thereafter the child and parent completed an AE checklist. The AE checklist was adapted from Kutcher [20]. The checklist was reviewed with the child and parent by the treating physician to determine frequency, severity, impairment, and any additional treatment. Any SAE was reported to the principal investigator (TI). Details of all the measures used in NordLOTS are described elsewhere [45].

### Interventions

#### Sertraline

Sertraline treatment included 6 sessions over 16 weeks. Sertraline was chosen because it is the only approved SSRI for OCD treatment in children and adolescents in Denmark, Sweden and Norway [45] and it has shown to be equally effective as the other SSRIs [1]. The pharmacotherapy treatment manual was adapted from the manual used in the POTS study [30]. A starting dose of 25 mg per day was titrated up to 100 mg per day by week 4; children below 10 years of age with low weight could be started on a lower dose, if deemed necessary. If response was considered inadequate at a dose of 100 mg, the dose was increased gradually up to a maximum of 200 mg per day by week 8. Treatment response and AEs were monitored at every visit and the dose reduced if necessary. The manual also included guidelines for clinical support where participants were encouraged to practice exposure tasks learned during Step 1 treatment. However, introducing new exposure tasks was not allowed. The overall rationale for this component was to reduce the variability of non-specific treatment effects unrelated to SRT. Pharmacotherapists were required to use a standardized script and were instructed to (1) ask child and parent(s) about resistance to compulsions; (2) review activities the child will engage in once he or she improves; (3) whether the child actively uses treatment techniques learned during Step 1; (4) whether the child has engaged in exposures and the resulting outcome; (5) encourage the child to continue to do exposures and to not engage in avoidance or rituals; and (6) inquire and address any concerns the family has related to the medication.

#### Continued CBT

Participants randomized to continued CBT received 10 additional treatment sessions over 16 weeks. The same CBT principles used in Step 1 were used in Step 2. However, the therapist needed to conduct a new case formulation by identifying barriers that may have interfered with treatment received in Step 1. Common factors identified were: (1) the patient took long time to engage and start exposure exercises due to high levels of anxiety or low motivation; (2) family factors, such as high initial accommodation which made exposure homework difficult; (3) OCD appeared predominantly at home, school or other places and in-office exposures did not generate high enough anxiety levels to be helpful; (4) the participant’s own expectation for improvement was low and less effort was exerted during exposure exercises; (5) a non-optimal relationship with the therapist may have interfered with treatment motivation. Based on these and other factors, the Step 2 CBT was tailored to address specific problems as deemed clinically appropriate by the treatment team. For instance, Step 1 participants whose onset of exposure exercises was delayed, for whatever the reason, were simply given more exposure exercises in Step 2 CBT. Or, for Step 1 participants whose OCD symptoms occurred primarily at home or at school, steps were taken to conduct in vivo exposures in the setting in which the symptoms most frequently occurred. Or, if certain family dynamics were seen as counter-productive, measures were taken to address these barriers. For instance, helping parents to not accommodate to their child’s OCD, decreasing OCD-related blame and guilt, or helping to generate developmentally appropriate patterns of family interactions. For these reasons, the Step 2 CBT was less manualized and more individually tailored to the needs of the participant while staying consistent with principles of good E/RP CBT. The format of sessions in Step 2 CBT was the same as in step 1 [47]. Sessions were scheduled for 90 min, where the first hour centered on individual E/RP and the last half hour included family sessions with one or both parents. The family sessions were conducted in a similar manner as described by Piacentini and colleagues [28].

### Statistical analysis

Of the 50 participants who entered Step 2 treatments, 14 (28 %) participants withdrew consent (seven in each treatment condition). However, all randomized participants who had a CY-BOCS total score of ≥16 before Step 2 treatment started were included in the analyses according to intent-to-treat principles.

The statistical method employed was piecewise regression [38], sometimes referred to as a longitudinal discontinuity model [40]. This model, herein called the longitudinal discontinuity model, evaluates whether a shift in the outcome trajectory occurs following the occurrence of a known event. In this paper, the known event is the onset of Step 2 treatment. That is, the longitudinal discontinuity model was used to evaluate whether the introduction of sertraline in CBT non-responders would impact the symptom trajectory more than continuing CBT.

In order to evaluate longitudinal discontinuity models, a point in time along the trajectory is specified that marks the onset of when a given treatment was introduced. A significant change in slopes following the onset of Step 2 treatments suggests that the newer treatments are having an impact on the Step 2 outcomes above what would be expected if no treatment had been provided.

To address how sertraline could affect a participant’s outcome trajectories, we conducted a standard multilevel model for change (or mixed effects linear regression) that included two random effects (intercept and days since baseline) and the following fixed effects: binary indicators for site ([grand mean centered] Aarhus, Southern and Eastern Norway, Central Norway, Stockholm), with Gothenburg serving as the reference category. To this basic model, a series of discontinuous multilevel models for change were fitted to the data using restricted maximum likelihood estimation. The outcome modeled (CY-BOCS total score; COIS-R) varied by the hypothesis under consideration and was evaluated by introducing to the “baseline” model a second level-1 individual growth trajectory with a discontinuity in both elevation and slope that marked the onset of Step 2 treatment: namely, sertraline. The variable TREATMENT, which was allowed to vary across subjects, was used to mark the onset of Step 2 treatments. This variable can be thought of as a new intercept for the second level-1 individual growth trajectory, and if found to be statistically significant, suggests that the addition of sertraline resulted in a shift (either up or down) in the average outcome trajectory. The variable POSTDAYS, also a time-varying predictor, marked the passage of time following receipt of sertraline. This variable, if found to be statistically significant, suggests that the average slope (i.e., rate of change) following receipt of sertraline was different than the slope during the period preceding the onset of Step 2 treatments. In other words, it captures the additive effect of sertraline on the outcome trajectory after receipt of a given treatment.

In addition, to evaluate whether the discontinuity varied across participants (i.e., the magnitude of the discontinuity was not the same for all participants), models in which DAYS and POSTDAYS were treated as random effects (in addition to fixed effects) were evaluated. To evaluate whether the addition of random effects for these two parameters resulted in a better fitting model, the change in the deviance statistics relative to the baseline model was used. This difference follows a Chi-square distribution with degrees of freedom equal to the difference in the number of parameters between the two models. In all models, residual error terms were assumed to follow a mean-zero, normal distribution with an unstructured covariance structure used to capture the within person correlation over time. Tests were two-tailed, and a *p* value of less than .05 was considered statistically significant. Models were fit using PROC MIXED in SAS Statistical Software, Version 9.4 (SAS Institute, Cary, NC, USA).

Multivariate *χ* ^2^ tests were conducted on the binary outcome following 16 weeks of Step 2 treatment. Multiple imputation was used to replace missing data. This was done with a sequential regression multivariate imputation algorithm [36]. The imputation model included all outcome measures, time in weeks, treatment indicators, stratification variables (sex and tic disorder) and all possible predictors and moderators. A total of 200 data sets were generated in accordance to recent guidelines [6, 16] in order to make both estimates, confidence intervals and p values reliable. Outcomes reported were calculated using Rubin’s rules [36] for combining the results of the 200 identical analyses. This was done on each of the 200 imputed data sets and the results were combined and reported as an *F* statistic. Tests were two-tailed, and a *p* value of less than 0.05 was considered to indicate statistical significance. Multiple imputation was conducted by using the SAS macro IVEware [31], and computation of a combined *F* statistic was conducted with the SAS macro COMBCHI [3].

We performed a power analysis on the adjusted post-treatment CY-BOCS total score which based on simulation in the multilevel models of each observation of the CY-BOCS. Sample size was adequate to detect a moderate effect size (*d* = 0.50). We consider this to reflect a clinically significant treatment effect and correspond to a mean difference on the CY-BOCS of five points.

## Results

### Recruitment and retention

**Fig. 1:**
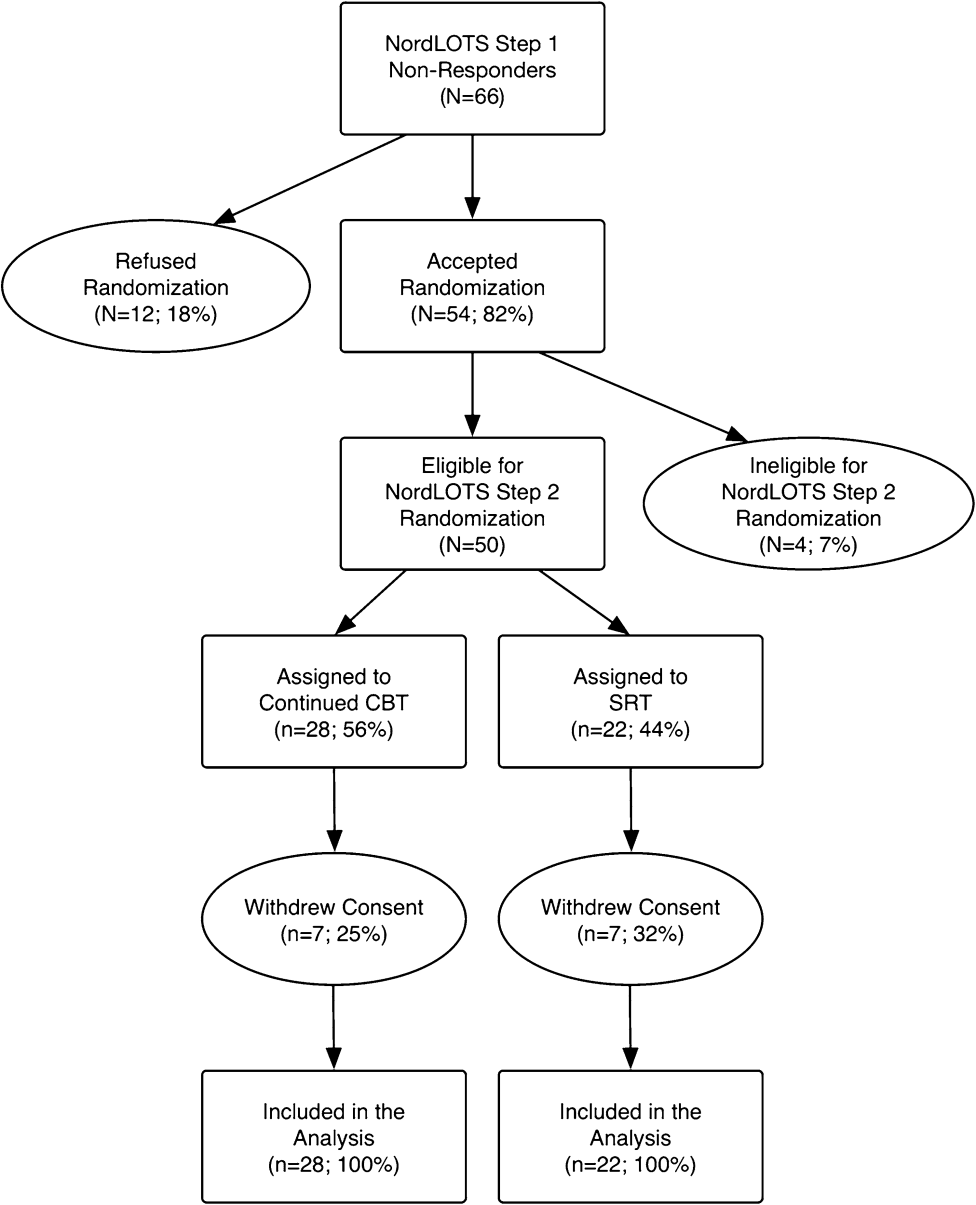
CONSORT flow diagram of the NordLOTS Step 2

A total of 54 participants were randomized to SRT or continued CBT. Four participants assigned to SRT were re-evaluated using the CY-BOCS due to a treatment delay of more than 3 weeks. These four participants scored below 16 on the CY-BOCS reassessment and were therefore considered Step 1 treatment responders and not eligible for Step 2 treatment. Thus, they were not included in the Step 2 ITT sample. There were no significant differences between treatment condition in the percentage of participants who dropped out (i.e., withdrew consent) of the study, *χ* ^2^(1, 49) = 0.046, *p* = .830 (CBT = 25 %; SRT = 32 %). All SRT participants who dropped out did so because they were opposed to medication use for pediatric OCD. Six participants randomized to continued CBT did not wish to continue with CBT, while one participant terminated treatment because of somatic disease. For the remaining participants, treatment adherence for CBT was adequate, with 61.9 % (*n* = 13) of participating children showing “good” or “very good” levels of compliance and 95.2 % (*n* = 20) for CBT parents. In SRT, 53.3 % (*n* = 8) of participating children showed good or very good compliance and 90.5 % (*n* = 19) for SRT parents. The CONSORT flow diagram for the trial is shown in Fig. 1.

### Patient characteristics

Step 2 participant characteristics and assessments are reported in Table 1. The mean CY-BOCS total score reduction in percentage from baseline to week 13 was 16.4 (SD = 19.8). Sixteen participants (32.0 %) had elevated CY-BOCS scores or a reduction in the CY-BOCS of less than 10 % from baseline to week 13. Twenty-one (42.0 %) had a moderate reduction in the CY-BOCS of less than 30 % from baseline to week 13, while 13 (26.0 %) showed 30–47 % reduction in the CY-BOCS over this same period. No statistically significant difference was revealed between the groups *t*(48) = −1.01, *p* = .317. Step 1 CBT treatment adherence was adequate, as 48 % (*n* = 24) of the participating children showed “good” or “very good” levels of compliance and 76 % (*n* = 38) of the parents.

**Table 1:** Baseline demographic and clinical characteristics by treatment group in NordLOTS Step 2

Characteristics	Sertraline (*n* = 22)	CBT (*n* = 28)	Total (*n* = 50)
Sex [No. (%)]			
Male	11 (50.0)	13 (46.4)	24 (48.0)
Female	11 (50.0)	15 (53.6)	26 (52.0)
Age [mean (SD) in years]	14.1 (2.8)	14.0 (2.8)	14.0 (2.7)
Family status [No. (%)]			
Biological parents living together	15 (68.2)	16 (57.1)	31 (62.0)
Divorced	7 (31.8)	12 (42.9)	19 (38.0)
SES [No. (%)]			
High	14 (63.6)	16 (57.1)	30 (62.5)
Low	8 (36.4)	10 (35.7)	18 (37.5)
Ethnicity [No. (%)]			
At least one Scandinavian parent	22 (100)	27 (96.4)	49 (98.0)
Scalar variables [mean (SD)]			
CY-BOCS total score week 0	25.2 (5.0)	27.3 (5.9)	26.4 (5.6)
CY-BOCS total score week 13	21.1 (3.7)	21.3 (4.0)	21.3 (3.8)
CGAS week 0	52.8 (10.3)	52.3 (6.4)	52.5 (8.2)
CGAS week 13	58.1 (10.5)	58.6 (8.2)	58.4 (9.2)
Psychiatric comorbid disorders [No. (%)]			
Any depressive disorders	0 (0)	3 (10.7)	3 (6.0)
Any anxiety disorders	4 (18.2)	8 (28.6)	12 (24.0)
ADHD	2 (9.1)	5 (17.9)	7 (14.0)
ODD and CD	0 (0)	1 (3.6)	1 (2.0)
Tic disorders	5 (22.7)	7 (25.0)	12 (24.0)
Any disorder	7 (31.8)	17 (60.7)	23 (46.0)

### Primary outcomes

**Fig. 2:**
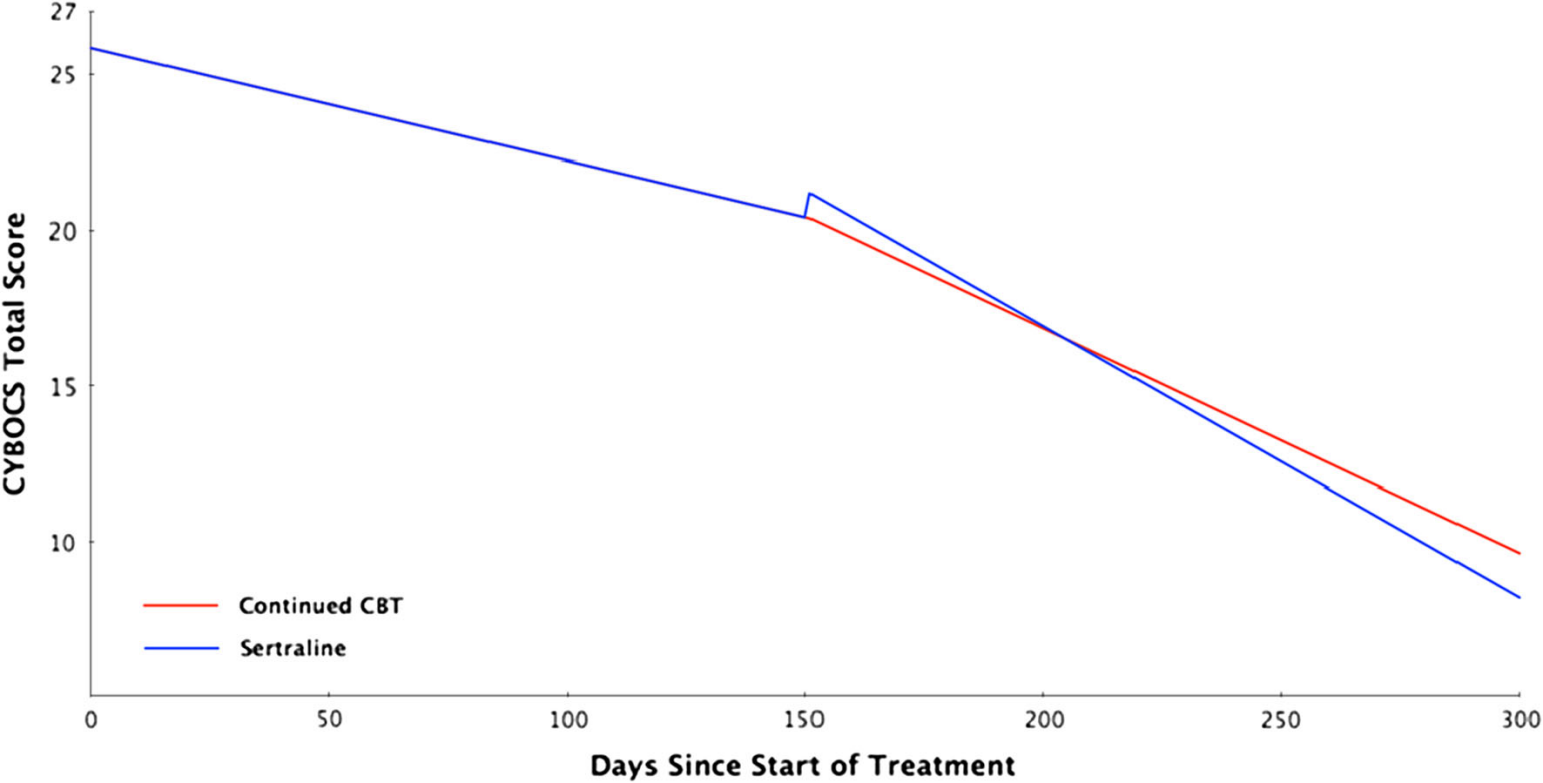
Adjusted Intent-to-Treat CY-BOCS total score by days from baseline by treatment

Planned pairwise comparison at week 30 showed that the difference between continued CBT and SRT was not statistically significant *t*(119) = −0.94, *p* = .351 (See Table 2 and Fig. 2).

**Table 2:** Post-treatment Group-specific mean and response rates

	Estimated mean or rate (95 % CI)^a^		Effect sizes CBT vs. SRT (95 % CI)^b, c^
	Continued CBT	Sertraline	
Primary outcomes			
CY-BOCS total score^d^	13.64 (10.96–16.32)	11.65 (7.88–15.42)	−0.29 (−0.85 to −0.27)
CY-BOCS <16^e^	0.50 (0.33–0.67)	0.45 (0.27–0.65)	0.10 (−0.72 to 0.52)
Secondary outcomes			
COIS-R parent report^f^	17.20 (11.74–22.67)	15.50 (8.86–22.14)	−0.11 (−0.67 to 0.45)
COIS-R child report^f^	15.39 (11.05–19.73)	8.44 (2.72–14.17)	−0.55 (−1.12 to −0.02)
CY-BOCS <11^g^	0.32 (0.18–0.51)	0.27 (0.13–0.48)	−0.22 (−0.41 to 0.85)
30 % reduction^h^	0.36 (0.21–0.54)	0.45 (0.27–0.65)	0.13 (−0.81 to 0.55)

^a^For CY-BOCS total score and COIS-R estimated mean score at week 30 from the fitted multilevel model. For the responder status, the estimated rate of response at week 30

^b^For CY-BOCS total score and COIS-R, between-groups difference in estimated mean score at session 30. For the responder status, between-groups difference in rate at week 30

^c^Negative effect size suggests that SRT was more effective and positive effect size suggests that continued CBT was more effective

^d^CY-BOCS total score range from 0 to 40 with larger scores reflecting more OCD symptoms

^e^CY-BOCS <16 reflects participants that obtained CY-BOCS total score of 15 or below at week 30

^f^COIS-R total score range from 0 to 99 with larger scores reflecting more OCD-related functional

^g^ CY-BOCS < 11 reflects participants that obtained CY-BOCS total score of 10 or below at week 30

^h^30 % reduction of CY-BOCS total score from week 13 to week 30

The binary CY-BOCS total score (CY-BOCS <16) showed that 48.0 % (*n* = 24) of participants across both treatment conditions were rated as treatment responders at week 30. In the continued CBT group, 50 % (*n* = 14) were deemed treatment responders at week 30 and 45.4 % (*n* = 10) in the SRT group. The multivariate *χ* ^2^ test suggested that there were no statistically significant differences between continued CBT and SRT in the proportion of treatment responders (*p* = .727).

### Secondary outcomes

On the parent COIS-R, planned pairwise comparison at week 30 showed that the difference between continued CBT and SRT was not statistically significant (*p* = .660). However, on the child COIS-R, the SRT group had reported statistically significant lower scores when compared to the continued CBT group, *t*(89) = −2.08, *p* = .040 (see Table 2). A total of 40 % of all participants showed 30 % (*n* = 20) or more reduction on the CY-BOCS total score from week 13 to week 30 (Step 2 treatment period). In the continued CBT group, 35.7 % (*n* = 10) showed a 30 % or greater reduction on the CY-BOCS total score from week 13 to week 30, and 45.4 % (*n* = 10) in the SRT group. The difference was not statistically significant (*p* = 0.476). A total of 30 % (*n* = 15) of all Step 2 participants had a CY-BOCS total score of 10 or below at post-treatment (week 30). In the continued CBT group, 32.1 % (*n* = 9) had CY-BOCS below 11 at week 30 assessment point and 27.2 % (*n* = 6) in the SRT group achieved a CY-BOCS total score of 10 or below. This difference was not statistically significant (*p* = .676).

### Effect estimates

The within-group effect size [24] for CY-BOCS total score was 1.19 (95 % CI = 0.54–1.83) for the SRT group and 1.04 (95 % CI = 0.47–1.61) for continued CBT. The effect size for the parent COIS-R was 0.45 (95 % CI = −0.16 to 1.06) for the SRT group and 0.39 (95 % CI = −0.14 to 0.92) for continued CBT. The effect size for the child COIS-R was 0.85 (95 % CI = 0.22–1.47) for the SRT group and 0.24 (95 % CI = −0.29 to 0.77) for the continued CBT group.

### Adverse events

A total of 12 of 15 SRT participants (80.0 %) reported at least one mild adverse event (AE) during the Step 2 treatment. AE outcomes are shown in Table 3. The most frequent AEs were gastrointestinal. Two participants reported mild increased suicidal ideation and one reported new onset mild suicidal thoughts. However, these events were attributed to current life stressor(s)/situation rather than the medication; and, hence, the participants did not discontinue their study medication. No SAEs were reported.

**Table 3:** Adverse events in SSRI-treated participants

	*n* (%)
Participants with AE ≥1	12 (80.0)
Gastrointestinal	11 (73.3)
Psychiatric other than suicidal	9 (60.0)
Suicidal thoughts or ideation	3 (20.0)
Sleep	2 (13.3)
Autonomic	6 (40.0)
Neurological	5 (33.3)
Menstruation	1 (6.7)
Sexual	1 (6.7)
Skin problems	1 (6.7)

## Discussion

The objective of the study was to compare sertraline with continued CBT for the treatment of pediatric OCD among a sample of children who had received an adequate trial of CBT (14 weeks of individual outpatient exposure-based CBT) and deemed to be CBT non-responders (CY-BOCS ≥16).

RCTs of treatment-resistant pediatric OCD are extremely rare [12, 17], especially given the low prevalence of OCD and the success rate of first-line treatments [28, 30, 47]. This is the first RCT of CBT non-responders in pediatric OCD to date. The study is not a direct comparison of CBT and SSRI in a sample of treatment naive children and adolescents. Rather, it is a comparison between CBT and SSRI among children and adolescents with OCD who failed to respond or benefit from an initial course of CBT.

Overall, there were no significant differences on the primary outcome measures between SRT and continued CBT among children who did not show an adequate response to an initial course of CBT. However, within-group effect size estimates on the CY-BOCS total score were large for both continued CBT (1.04, 95 % CI = 0.47–1.61) and SRT (1.19, 95 % CI = 0.54–1.83) and the response rate across both groups was 48.0 %. The response rate in step 1 was 72.6 % [47] and the combined Step 1 and Step 2 response rate was 81.4 %, indicating, that improvement can still be achieved with continued treatment. The rate of remitters (CY-BOCS <11) was 30.0 % across groups in this trial. In step 1 it was 49.4 % [47] and the combined Step 1 and Step 2 remittance rate was 55.0 %. Although the Step 1 remittance rate was already high (higher than reported in previous CBT trials [28, 30]), response rates in children that initially received CBT continued to increase regardless of whether they continued CBT or switched to SSRI.

Analyses of the child COIS-R showed that the SRT group had significantly lower scores compared to the continued CBT group, while there were no significant differences on the parent COIS. Children in the SRT group reported less OCD-related functional impairment at post-treatment than the continued CBT group. The within-group effect size estimate for the SRT group was large (0.85, 95 % CI 0.22–1.47) and statistically significant, while the within-group effect size estimate for the continued CBT group was small (0.24, 95 % CI −0.29 to 0.77) and non-significant. No clinically meaningful cut-off points have been developed for the COIS-R and the test–retest variability of the scale has not been established. However, the effect size estimate for the SRT group is similar to results reported in previous studies [28, 42].

The results of the primary outcome measures in the current study stand in contrast to the adult OCD study where fluvoxamine proved to be superior to cognitive therapy without E/RP [51]. In that study, participants randomized to CBT did not continue with E/RP in Step 2, but switched to cognitive therapy without E/RP which was limited to twelve 45-min sessions. Previous studies on adults with OCD have produced mixed results in terms of effectiveness of cognitive therapy without E/RP, some have not shown any difference [8, 52] and other have shown that E/RP is superior [9, 27]. One speculation is that our continued individual exposure-based CBT may have produced more effect than the cognitive therapy used in the adult OCD study.

The CBT in Step 2 had the same format as Step 1 CBT. Sessions were scheduled for 90 min, the first 60 min centered on individual E/RP while the last 30 min included family sessions with one or both parents. In spite of a reassessment and new case formulation prior to its initiation, step 2 CBT did not differ considerably from Step 1 CBT in frequency, intensity, format, etc. The large effect size of Step 2 CBT and additional response rate from Step 1 response indicates that children with OCD that do not respond to initial CBT may benefit by engaging in CBT for a longer period given that a new case formulation with identification of interference factors is implemented.

The side effect profile of sertraline in the current study was similar to those reported in other studies [21, 30]. It should be noted that no SAEs were reported. AEs of the participants randomized to continued CBT were not assessed, so it is not possible to compare differences between the two groups or estimate the base rate of AEs among participants who receive CBT. However, the results suggest that SRT is well tolerated as no participant needed to be prematurely terminated from treatment because of AEs. These findings mirror those reported in the literature for acute and 1-year outcomes [7, 53].

Results from the current analyses cannot be used to inform clinical decisions regarding which treatment (SRT or continued CBT) should be used following first-line CBT treatment. Current choices for a non-responding patient after 14 weeks would be: (1) to continue on CBT, optimally adjusted by a new case formulation or (2) switch to an SSRI. Other choices not evaluated in this study would be to combine CBT and SSRI. This choice is not strongly supported by empirical data. No studies exist on CBT non-responders. The POTS study suggested the superiority of combined treatment (POTS, [30]). In contrast, a recent study by Storch and colleagues [42] did not find a significant differences between CBT and pill placebo versus CBT and sertraline. Another possibility for CBT non-responders would be to adapt the CBT, for instance by intensifying it (increasing session length and session frequency). Two uncontrolled trials have reported promising results in treatment-resistant children and adolescents with OCD [5, 43].

### Strengths and limitations

This is the first methodologically rigorous randomized controlled trial of CBT non-responders in pediatric OCD. A comprehensive standardized assessment battery has been employed, specifically trained and experienced independent evaluators and two active treatment comparisons were used.

Despite these strengths, there are limitations to the study. Although the severity of OCD continued to decrease in Step 2, it remains unclear how much of this decrease can be attributed to the treatments offered in Step 2 or to possible carry-over effects from Step 1. In this trial treatments were not compared to no treatment or placebo controls. Thus, the absolute effect of sertraline or continued CBT is not known. A future trial with a placebo control condition will be needed to rule out possible carry-over effects.

The attrition rate was fairly high, 28 % across both treatment groups. All the SRT participants dropped out because parents did not wish medication as a treatment for OCD symptoms in their children while only one continued CBT participants did not want to continue CBT. The retention between steps seems to be a common problem in stepped care studies [15, 46].

## Conclusion

Results did not show statistically significant differences between two treatment groups (continued CBT or SRT) among a sample of non-responders to CBT in pediatric OCD. However, within-group effect size estimate were large and significant across both treatments, suggesting that CBT non-responders are likely to benefit from either continued CBT or switching to an SSRI.
